# FTO in cardiovascular diseases: mechanisms, context dependence, and translational opportunities

**DOI:** 10.3389/fcell.2026.1850701

**Published:** 2026-05-20

**Authors:** Yifan Kong, Di Zhang

**Affiliations:** 1 School of Healthy Aging, Shandong Women’s University, Jinan, China; 2 Rehabilitation Medicine Research Office, Shandong Provincial Third Hospital, Jinan, China

**Keywords:** cardiovascular diseases, context-dependent regulation, epitranscriptomics, FTO, RNA methylation

## Abstract

Cardiovascular diseases (CVDs) remain the leading cause of death worldwide. Their regulation involves not only classical genetic mechanisms but also dynamic epitranscriptomic control. The fat mass and obesity-associated protein (FTO), an N^6^-methyladenosine (m^6^A) RNA demethylase, has been implicated in cardiovascular disease. Evidence shows that the role of FTO in CVDs is strongly context dependent, with both protective and harmful effects reported in different settings. This review summarizes the genetic, molecular, and epitranscriptomic features of FTO and presents a framework in which FTO acts through three connected axes: metabolic remodeling, immuno-inflammatory signaling, and electrophysiological and structural remodeling. By regulating key transcripts through RNA methylation-related post-transcriptional control, FTO may modulate cellular responses to hypoxia, inflammation, and metabolic stress. It also reviews the context-specific roles of FTO in atherosclerosis, hypertension, myocardial infarction, ischemia-reperfusion injury, myocardial fibrosis, heart failure, arrhythmia and myocarditis. These different effects seem to depend on cell type, target selection, and disease stage, which suggests that FTO acts as a context-sensitive epitranscriptomic switch rather than a simple one-way effector. FTO represents a promising but complex therapeutic target. Pharmacological inhibition of FTO has shown benefit in some disease settings, but other studies suggest that selective activation or context-dependent modulation may also be needed. However, the precise biochemical functions of FTO and the relative contributions of RNA modifications remain incompletely understood. Key barriers include limited causal evidence, poor cell-specific resolution, and incomplete integration with other epigenetic layers.

## 1 Introduction

Cardiovascular disease (CVD) includes a wide range of disorders of the heart and blood vessels and remains the leading cause of death worldwide (Kaminsky et al., 2022). Genetic and epigenetic studies have greatly improved our understanding of CVD pathogenesis and have pointed to new targets for prevention and treatment.

Disease-associated variation can be examined at the gene and protein levels. Fat mass and obesity-associated protein (FTO) has been implicated in the pathogenesis of multiple disorders, including CVDs (Jiao et al., 2026; Kazarnovsky Nahshan et al., 2026; Wang et al., 2026). *Fto* was initially identified in 1999 as one of several genes deleted in mice carrying the Fused toes (*Ft*) mutation, although its function was unclear at that time (Benak et al., 2024c). Subsequent genome-wide association studies (GWAS) in 2007 demonstrated that single-nucleotide polymorphisms (SNPs) in the human *FTO* gene are associated with increased body mass index (BMI) and obesity (Frayling et al., 2007). Early studies showed that several SNPs in the first intron of *FTO* are significantly associated with obesity in humans (Loos and Yeo, 2014). The relationship between *FTO* SNPs and *FTO* expression remains controversial (Dina et al., 2007; Klöting et al., 2008; Stratigopoulos et al., 2008; Berulava and Horsthemke, 2010), but animal studies clearly show that *Fto* deficiency causes growth retardation with marked reductions in adipose mass and body weight, which points to a key role for *Fto* in adipogenesis, adipose tissue maintenance, and body-weight regulation (Merkestein et al., 2015). Beyond obesity, *FTO* polymorphisms have also been associated with insulin resistance, metabolic syndrome, atherosclerosis, and systemic hypertension. (Zhang et al., 2023; Song et al., 2025; Yang et al., 2025; Zhou et al., 2025; Xiao et al., 2026). Variants in *FTO* have also been linked to cardiovascular events such as hypertension, myocardial infarction (MI), and acute coronary syndrome (ACS) (Takeuchi et al., 2023; Xu et al., 2023; Lin et al., 2025).

RNA modification has become a key area of CVD research and offers new opportunities to clarify disease mechanisms and develop novel therapeutic strategies. Subsequent studies identified FTO as an RNA demethylation-related enzyme involved in the regulation of N^6^-methyladenosine (m^6^A), one of the most prevalent internal RNA modifications involved in multiple fundamental physiological processes (Jia et al., 2011). Accumulating evidence suggests that FTO-dependent m^6^A demethylation may contribute to the initiation and progression of several CVDs (Wang et al., 2024; Yu et al., 2025; Zhu et al., 2025) and is closely associated with hypertrophic cardiomyopathy, congenital heart defects, heart failure (HF), and coronary heart disease (CHD) (Kuveljic et al., 2024; Jing et al., 2025; Min et al., 2025; Tan et al., 2025).

FTO-mediated RNA demethylatio has multiple effects in CVDs, and these effects are strongly context dependent. Studies have reported both protective and harmful outcomes in different disease settings. This pattern suggests that FTO acts as a dynamic regulator across metabolic, inflammatory, electrophysiological, and structural pathways rather than as a uniformly harmful or protective factor.

This review provides an overview of the genetic and epitranscriptomic features of FTO, presents a conceptual framework for its cardiovascular actions, and discusses its context-dependent roles across major CVDs. It also points out current controversies, methodological limits, and the possible value of FTO as a biomarker and therapeutic target.

## 2 Molecular and epitranscriptomic basis of FTO in cardiovascular systems

*FTO* found in various tissues such as adipose, heart, and brain, but its expression is highest in the brain, especially in hypothalamic nuclei known to control energy balance (Gerken et al., 2007). In humans, the *FTO* gene is located on chromosome 16q12.2, spans 410.50 kb, and contains nine exons and eight introns. The full-length FTO protein comprises 505 amino acids and includes two major domains: an N-terminal domain (NTD; residues 1–326) and a C-terminal domain (CTD; residues 327–505) (Han et al., 2010).

### 2.1 Genetic architecture of *FTO* and its association with cardiovascular diseases

SNPs are the most common form of genetic variation and account for approximately 90% of sequence variability in the human genome (International et al., 2007). Depending on their location, SNPs can influence disease susceptibility by altering protein structure or gene expression. Evidence suggests that variation in *FTO* is associated with CVD risk. *FTO* variants are predominantly located in noncoding regions, particularly within the first intron, and may indirectly contribute to CVD development by increasing cardiometabolic risk factors such as obesity, diabetes, and chronic inflammation (Berulava and Horsthemke, 2010; Eghbali et al., 2026). Some studies further suggest that *FTO* may participate more directly in the disease processes of hypertension, ischemic cardiomyopathy, and HF, representing a potential causal factor as well as a therapeutic target in CVD (Zhang et al., 2025).

Genetic variation is a major contributor to CVD susceptibility. Previous studies show that *FTO* variants are associated with increased risks of MI, ACS, and heart-transplant rejection (Hubacek et al., 2018; Takeuchi et al., 2023; Fan et al., 2025; Janzi et al., 2025; Lin et al., 2025). Certain heterozygous or homozygous risk variants identified in human genetic studies, including European population-based cohorts (e.g., HAPIEE) and clinical cohorts of cardiovascular patients, may be associated with reduced *FTO* expression, increasing the risk of CHD and ACS (Hubacek et al., 2018). One study based on two Swedish population-based case-control cohorts (INTERGENE and SHEEP) reported that the male-to-female ratio of *FTO* mutation carriers was 1.17 among randomly selected population controls, but increased to 2.47 among patients with chronic heart disease (Gustavsson et al., 2014). In addition, specific *FTO* variants are strongly associated with type 2 diabetes mellitus (T2DM) and obesity, both of which are established risk factors for CVD (Dina et al., 2007; Frayling et al., 2007; Fragoso-Bargas et al., 2025; Köksal et al., 2026). GWAS data further indicate that risk variants in the first intron of *FTO* are closely linked to elevated BMI and increased obesity risk (Frayling et al., 2007), and in a Brazilian case-control cohort of individuals with extreme obesity, homozygous carriers of the risk allele tend to have greater body weight and a substantially higher risk of obesity (Salum et al., 2025).

Multiple SNPs in *FTO*, including rs1421085, rs17817449, rs9930506, and rs9939609, have been associated with obesity in both children and adults, as well as with related metabolic phenotypes (Dina et al., 2007; Groop, 2007; Scuteri et al., 2007; Loos and Yeo, 2014). A GWAS of fat distribution found that *FTO* is more strongly associated with subcutaneous fat than with visceral fat (Fox et al., 2012), which suggests that its biological effects may not fully overlap with those of the classical insulin-resistance pathway (Moon et al., 2018; Zhang et al., 2024). Across multiple populations, including European ancestry cohorts, Chinese Han populations, South Asian populations, and Finnish cohorts, rs8050136, rs9939609, rs17817449, and rs12149832 have all been associated with BMI or CVD risk (Ahmad et al., 2010; He et al., 2010; Lappalainen et al., 2011; Ningombam et al., 2018; Gu et al., 2020b; Mahmoud et al., 2022; AlAnazi et al., 2024). Some SNPs may act by altering transcription-factor binding or by regulating the expression of *FTO* or nearby genes. For example, rs1421085 disrupts binding of the AT-rich interactive domain-containing protein 5B (ARID5B) repressive complex and derepresses iroquois homeobox 3 (*IRX3*) and iroquois homeobox 5 (*IRX5*) (Claussnitzer et al., 2015), which are linked to cardiac impulse conduction, cardiac remodeling, and cardiac dysfunction (Postma et al., 2011; Zhang et al., 2011).

Although substantial evidence supports a link between *FTO* variants and increased CVD risk, these effects are not entirely BMI dependent. In population-based studies from Denmark and Finland, the associations of rs8050136 and rs9939609 with CVD risk or CVD-related mortality persisted after adjustment for BMI (He et al., 2010; Lappalainen et al., 2011; Borglykke et al., 2012; Äijälä et al., 2015). By contrast, a study in an Iranian population found no significant association between rs9939609 and CHD (Mofarrah et al., 2016). Such discrepancies may reflect differences in ethnicity, lifestyle, environmental exposure, and sample composition. SNPs may also influence m^6^A modification sites by altering RNA sequences; previous studies have identified many m^6^A-SNPs, some of which are associated with coronary artery disease (Mo et al., 2018; Qin et al., 2020). *FTO*-associated variants may influence CVD through metabolic phenotypes, BMI-independent mechanisms, or both pathways simultaneously.

**FIGURE 1:**
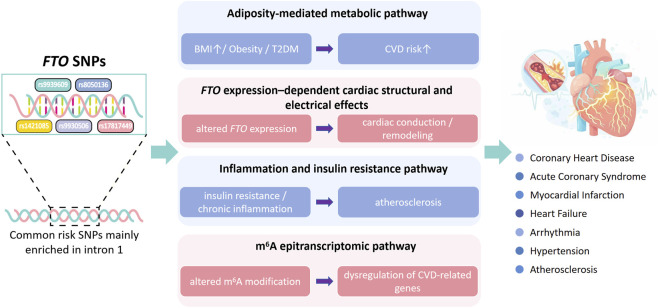
Mechanistic links between FTO genetic polymorphisms and cardiovascular diseases.

*FTO* SNPs may affect CVD susceptibility by changing the expression of *FTO* or related genes (Figure 1). Still, association studies alone cannot prove causality, and more functional work is needed to show how these variants drive cardiovascular injury.

### 2.2 Epitranscriptomic function of FTO in m^6^A regulation

RNA epigenetic modifications, particularly m^6^A methylation, have attracted extensive attention in oncology, metabolic disease, and cardiovascular research. Increasing evidence indicates that epigenetic and transcriptomic regulation are closely involved in the onset and progression of CVD (Zhong et al., 2016; van der Harst et al., 2017; Agha et al., 2019; Kuznetsova et al., 2020; Li et al., 2020). m^6^A is one of the most abundant reversible chemical modifications in eukaryotic messenger RNA (mRNA) and plays a critical role in organ development, cell growth, metabolic regulation, and intracellular signaling (Fu et al., 2014; Yue et al., 2015; Mendel et al., 2018; Wen et al., 2018; Vu et al., 2019).

m^6^A is dynamically regulated by three classes of proteins: writers, erasers, and readers (Liu et al., 2019; Shi et al., 2019). Among the erasers, FTO and AlkB homolog 5 (ALKBH5) are recognized as the two major mammalian m^6^A demethylases. FTO belongs to the AlkB family and catalyzes oxidative demethylation in an Fe^2+^- and alpha-ketoglutarate (alpha-KG)-dependent manner (Gerken et al., 2007; Sanchez-Pulido and Andrade-Navarro, 2007; Jia et al., 2008). Jia et al. first demonstrated that FTO can effectively remove m^6^A from RNA, establishing the dynamic reversibility of m^6^A modification (Jia et al., 2011). In cancer, aberrant FTO activity regulates transcripts involved in proliferation, invasion, and apoptosis through m^6^A-dependent mechanisms (Li et al., 2019; Zhang et al., 2019). Similarly, dysregulated RNA modification has also been reported in cardiovascular conditions such as HF, stroke, coronary artery disease, and hypertension (Qiu et al., 2023).

FTO plays a key role in cardiovascular development and homeostasis. Loss of FTO function can lead to congenital heart defects and hypertrophic cardiomyopathy (Boissel et al., 2009). Altered *FTO* expression has been observed in patients with MI and HF as well as in corresponding animal models (Mathiyalagan et al., 2019; Shi et al., 2021; Zhang et al., 2021b; Vausort et al., 2022; Tu et al., 2024; Yang et al., 2024; He et al., 2025; Wang et al., 2025). In many pathological settings, reduced *FTO* expression is accompanied by elevated global m^6^A levels (Doaei et al., 2019; Abakir et al., 2020). This pattern may relate to the sensitivity of FTO, as an alpha-KG-dependent dioxygenase, to hypoxic or ischemic microenvironments: under physiological conditions, FTO is generally thought to recognize and remove methylated RNA marks, whereas under hypoxia its expression declines and its demethylase activity is impaired (Zhao et al., 2014). However, it should be noted that the precise biochemical function of FTO remains incompletely resolved. Recent evidence suggests that FTO may act not only as a demethylase but also as an RNA hydroxylase, thereby complicating the traditional view of FTO as a classical m^6^A eraser (Kaur et al., 2025).

Changes in the m^6^A status of key transcripts have been proposed to influence cardiac function. *In vitro* studies suggest that increased *Fto* expression promotes hypertrophy in neonatal mouse cardiomyocytes, whereas small interfering RNA (siRNA)-mediated *Fto* knockdown attenuates this effect (Gan et al., 2013). Under excessive pathological stress, however, *Fto* deficiency is generally associated with a worse cardiac phenotype: mice with *Fto* defects show reduced ejection fraction and greater ventricular dilation after aortic constriction (Berulava et al., 2020), whereas *Fto* overexpression can improve cardiac dysfunction (Zhang et al., 2021a; Li et al., 2022a). In primary cardiomyocytes, *Fto* knockdown suppresses glycolysis and lowers adenosine triphosphate (ATP) levels (Zhang et al., 2021a). In endotoxemia models, inhibition of *Fto* is likewise associated with aggravated myocardial inflammation and dysfunction (Dubey et al., 2022). However, the extent to which these effects are mediated specifically through m^6^A demethylation remains uncertain. Recent studies have shown that modulation of *FTO* expression does not necessarily result in substantial global changes in m^6^A levels, suggesting that its effects on m^6^A may be limited or highly context-dependent (Nicholson et al., 2025; Stejskal et al., 2025). Therefore, the observed phenotypic changes associated with *FTO* may not be solely attributable to m^6^A regulation and could involve additional RNA modifications or alternative molecular mechanisms.

In addition to m^6^A, N^6^,2′-O-dimethyladenosine (m^6^A_m_) is another common adenosine modification formed by further methylation of 2′-O-methyladenosine (A_m_). FTO can remove not only m^6^A but also m^6^A_m_, affecting mRNA stability (Mauer et al., 2017). m^6^A_m_ is located near the mRNA transcription start site adjacent to N^7^-methylguanosine (m^7^G) (Wei et al., 1975; Bokar, 2005). Importantly, m^6^A and m^6^A_m_ are chemically similar and are not reliably distinguished by many commonly used analytical approaches, particularly antibody-based sequencing methods (Benak et al., 2024b). As a result, some biological functions previously attributed to m^6^A demethylation may in fact be mediated by m^6^A_m_, which complicates the interpretation of FTO-dependent effects. Some studies suggest that FTO may have a higher affinity for m^6^A_m_ than for m^6^A, although this substrate preference appears to depend on subcellular localization: in the nucleus, FTO preferentially targets m^6^A, whereas in the cytoplasm it more readily demethylates m^6^A_m_ (Wei et al., 2018; Relier et al., 2021). In addition, FTO can also act on N^1^-methyladenosine (m^1^A) in tRNA (Wei et al., 2018), indicating a broader substrate spectrum than initially recognized.

### 2.3 A unified framework of FTO-mediated regulation in cardiovascular diseases

Evidence suggests that the role of FTO in CVDs cannot be explained by a single-pathway model. FTO instead seems to act as a central epitranscriptomic regulator that coordinates several biological processes through m^6^A-dependent post-transcriptional control.

Current data suggest that the cardiovascular actions of FTO can be grouped into three connected functional axes.

1. Metabolic remodeling axis: FTO has been reported to influence cardiac energy metabolism, potentially through modulation of RNA methylation, including m^6^A, on transcripts involved in glycolysis, fatty-acid oxidation, and mitochondrial homeostasis. For example, FTO-dependent demethylation appears to enhance the expression of metabolic regulators such as phosphoglycerate mutase 2 (PGAM2), improving glucose utilization and ATP production in cardiomyocytes (Zhang et al., 2021a). Disruption of this axis may contribute to the metabolic inflexibility observed in HF and ischemic injury (Deng et al., 2021).
2. Immuno-inflammatory axis: FTO also participates in the regulation of inflammatory responses by influencing macrophage polarization, cytokine expression, and immune signaling pathways. In some contexts, FTO-mediated demethylation has been reported to promote anti-inflammatory macrophage activation and limits myocardial injury after infarction (Lin et al., 2025). In other settings, however, FTO may stabilize transcripts involved in lipid-driven inflammation (Yu et al., 2021), underscoring its bidirectional and context-dependent immunomodulatory role.
3. Electrophysiological and structural remodeling axis: FTO has been reported to influence cardiac electrophysiology and structural remodeling, potentially through m^6^A-dependent regulation of ion-channel genes, calcium-handling molecules, and fibrosis-related pathways. Regulation of targets such as potassium voltage-gated channel subfamily E member 1 (KCNE1), lysyl oxidase (LOX), and Sarco/Endoplasmic Reticulum Ca^2+^-ATPase 2a (SERCA2a) links FTO to arrhythmogenesis, atrial fibrosis, ventricular remodeling, and contractile dysfunction (Yang et al., 2024; Tan et al., 2025; Gong et al., 2026).

These three axes are closely linked and help explain why FTO can have different effects in different cardiovascular conditions.

## 3 Context-dependent roles of FTO across cardiovascular diseases

A key feature of FTO biology in CVD is its context dependence. In some settings, higher FTO seems protective, especially in HF, MI, and ischemia-reperfusion injury. In other arrhythmic, fibrotic, or inflammatory settings, higher FTO may worsen pathological remodeling. These differences likely reflect cell type, target transcript choice, disease stage, and the local microenvironment.

FTO is best viewed as a context-dependent regulator rather than a uniformly beneficial or harmful factor in CVD (Table 1).

**TABLE 1:** Roles and mechanisms of FTO in cardiovascular diseases.

Disease	FTO expression	Functional role	Key targets/Pathways	Net effect	Evidence	References
Atherosclerosis	Upregulated	Protective	PPAR-gamma/CD36; AMPK/ABCA1-ABCG1	Reduces lipid uptake and inflammation, limiting plaque formation	Animal/cell	Mo et al. (2017)
	Upregulated	Detrimental	STAT1/PPAR-gamma/NF-kappa B	Promotes macrophage activation and foam cell formation	Cell	Chedraui et al. (2016)
	Upregulated	Detrimental	KLF2/eNOS/VCAM-1/ICAM-1	Enhances endothelial inflammation and monocyte adhesion	Cell	Krüger et al. (2020)
Hypertension	Genetic variants	Detrimental	—	Increases hypertension susceptibility	Human	Deng et al. (2021), Falbová et al. (2022), Ke et al. (2022)
Myocardial infarction	Upregulated	Protective	JAK1/STAT3	Promotes anti-inflammatory polarization and metabolic reprogramming	Animal/cell	Lin et al. (2025)
	Upregulated	Protective	SERCA2a	Maintains calcium homeostasis and improves contractility	Animal/cell	Vausort et al. (2022)
Ischemia-reperfusion injury	Upregulated	Protective	YAP1	Reduces apoptosis and inflammation	Animal/cell	Zhu et al. (2020)
	Upregulated	Protective	Mhrt	Inhibits cardiomyocyte apoptosis	Animal/cell	Ju et al. (2021)
Myocardial fibrosis	Upregulated	Protective	—	Attenuates myocardial fibrosis	Animal	Timpson et al. (2009), Xi et al. (2013a)
	Upregulated	Protective	circCELF1/miR-636/DKK2	Inhibits fibroblast activation and migration, attenuating fibrosis	Animal/cell	García-Solís et al. (2016)
	Upregulated	Protective	PI3K/AKT/GLUT2; PPAR-gamma/RXR alpha	Improves metabolism and inhibits apoptosis, attenuating fibrosis	Animal/cell	He et al. (2014)
Heart failure	Downregulated	Detrimental	CALM1/SMYD1	Promotes heart failure progression	Animal/cell/human	Dubey et al. (2022)
	Upregulated	Protective	Mhrt/caspase-3/Bax/Bcl-2	Attenuates apoptosis and improves heart failure	Animal/cell	Ju et al. (2021)
	Upregulated	Protective	SERCA2a/RYR2/MYH6/7	Improves cardiac contractility and attenuates heart failure	Animal/cell/human	Tu et al. (2024)
	Downregulated	Detrimental	PGAM2/GLUT4	Reduces glycolysis and ATP production, aggravating heart failure	Animal/cell	Bokar (2005)
	Upregulated	Detrimental	PI3K/AKT	Promotes adverse remodeling associated with heart failure	Animal/human	He et al. (2025)
Arrhythmia	Downregulated	Protective	—	Increases arrhythmogenic susceptibility	Animal	Shen et al. (2021)
	Downregulated	Protective	—	Associated with increased atrial fibrillation risk	Human	Hubacek et al. (2016)
	Upregulated	Detrimental	KCNE1	Enhances IKs and shortens APD, thereby promoting atrial fibrillation	Animal/cell/human	Tan et al. (2025)
	Upregulated	Detrimental	LOX	Promotes atrial fibrosis and atrial fibrillation	Animal/cell/human	Nikpay et al. (2015)
Myocarditis	Downregulated	Protective	—	Reduces fatty acid-induced inflammatory injury	Animal/cell	Owens et al. (2004)
	Upregulated	Protective	—	Suppresses inflammatory signaling and myocardial inflammation	Animal/cell	Wei et al. (1975)

Abbreviation: ABCA1, ATP-binding cassette transporter A1; ABCG1, ATP-binding cassette transporter G1; AKT, protein kinase B; AMPK, AMP-activated protein kinase; APD, action potential duration; ATP, adenosine triphosphate; Bax, BCL2-associated X; Bcl-2, B-cell lymphoma 2; CALM1, calmodulin 1; CD36, cluster of differentiation 36; circCELF1, circular RNA CUGBP Elav-like family member 1; DKK2, dickkopf WNT signaling pathway inhibitor 2; eNOS, endothelial NO synthase; FTO, Fat mass and obesity-associated protein; GLUT2, glucose transporter 2; GLUT4, glucose transporter 4; ICAM-1, intercellular adhesion molecule 1; IKs, slow delayed rectifier potassium currents; JAK1, Janus kinase 1; KCNE1, potassium voltage-gated channel subfamily E member 1; KLF2, Kruppel-like factor 2; LOX, lysyl oxidase; Mhrt, myosin heavy-chain–associated RNA transcript; miR-636, microRNA-636; MYH6/7, myosin heavy chain 6/7; NF-kappaB, Nuclear factor kappa-B; PGAM2, Phosphoglycerate mutase 2; PI3K, phosphatidylinositol 3-kinase; PPARgamma, peroxisome proliferator-activated receptor gamma; RXR alpha, retinoid X receptor alpha; RYR2, ryanodine receptor 2; SERCA2a, Sarco/Endoplasmic Reticulum Ca^2+^-ATPase 2a; SMYD1, SET and MYND domain containing 1; STAT1, Signal transduction activator 1; STAT3, signal transducer and activator of transcription 3; VCAM-1, vascular cell adhesion molecule 1.

### 3.1 Atherosclerosis

Atherosclerosis is a chronic inflammatory vascular disease characterized by the progressive accumulation of lipids, inflammatory cells, and fibrous components within the arterial wall, ultimately leading to luminal narrowing and plaque formation. The conversion of lipid-laden macrophages into foam cells is one of the key events in lesion development.

Studies show that *FTO* regulates cholesterol accumulation in macrophage foam cells. Mo et al. reported that *Fto* overexpression reduces total cholesterol and low-density lipoprotein cholesterol (LDL-C) levels and alleviates atherosclerosis in apolipoprotein E (ApoE)-deficient mice (Mo et al., 2017). FTO reduces lipid uptake by inhibiting the peroxisome proliferator-activated receptor gamma (PPAR-gamma)/cluster of differentiation 36 (CD36) pathway and promotes cholesterol efflux through activation of AMP-activated protein kinase (AMPK), which suppresses foam-cell formation (Mo et al., 2017). In other studies, FTO promotes inflammatory signaling under some conditions by enhancing pathways related to signal transducer and activator of transcription 1 (STAT1), PPAR-gamma, or nuclear factor-kappa B (NF-kappa B) and by influencing macrophage polarization (Gu et al., 2020a). In endothelial cells, FTO has been reported to influence inflammatory responses, potentially through an m^6^A-dependent mechanism involving YTH N^6^-methyladenosine RNA binding protein 3 (YTHDF3) (Mo et al., 2022). FTO deficiency can also promote macrophage polarization toward the classically activated (M1) phenotype (Hu et al., 2019). These findings suggest that FTO also has context-dependent effects in atherosclerosis.

In addition to immune cells, abnormal proliferation and migration of vascular smooth muscle cells (VSMCs) are critical drivers of plaque progression and restenosis (Owens et al., 2004; Zhu et al., 2020). Although no significant differences in the prevalence of rs8050136 and rs9939609 were observed in atherosclerotic stroke (Song et al., 2016), *FTO* variants may still indirectly contribute to plaque formation by influencing homocysteine, triglycerides, BMI, and total cholesterol (Davis et al., 2014; Chedraui et al., 2016). *FTO* expression is also elevated in human vascular tissues and obese mice. Endothelial-specific *FTO* deficiency does not markedly affect obesity or dyslipidemia, but it can alleviate high-fat-diet-induced impaired glucose tolerance, insulin resistance, and hypertension (Krüger et al., 2020). These findings suggest that the role of FTO varies across vascular cell types, and further work is needed to define its impact on plaque stability and vascular repair.

### 3.2 Hypertension

An increasing number of studies have examined the relationship between *FTO* variants and hypertension risk. Several population-based studies have shown that the rs9939609 variant is associated with an increased risk of hypertension, and in some analyses this association appears to be BMI dependent (Timpson et al., 2009; Xi et al., 2013a; Xi et al., 2013b; He et al., 2014; García-Solís et al., 2016; Song et al., 2019). *FTO* polymorphisms may influence blood pressure both indirectly through obesity-associated hemodynamic and metabolic abnormalities and more directly through broader blood-pressure regulatory mechanisms.

Variants including rs9302652, rs17817449, rs8050136, and rs9926289 have also been associated with hypertension or related phenotypes (Pausova et al., 2009; Kumar et al., 2013; Falbová et al., 2022). For example, rs9302652 may be linked to enhanced sympathetic nervous-system regulation (Pausova et al., 2009), rs17817449 may be associated with gamma-glutamyltransferase levels and vascular remodeling (Falbová et al., 2022), and interactions between *FTO* and guanine nucleotide-binding protein subunit beta-3 (*GNB3*) variants may also influence the phenotype of essential hypertension (Kumar et al., 2013). However, some studies have found no significant association between *FTO* variants and systolic blood pressure in adolescents (Goulet et al., 2021). A meta-analysis including 57,464 patients with hypertension and 41,256 controls showed that *FTO* variants were associated with hypertension risk in both European and Asian populations; in Asian populations, this association persisted after adjustment for body weight (He et al., 2014). *FTO* variants may represent one component of hypertension susceptibility, although the underlying molecular mechanisms remain to be clarified.

### 3.3 Myocardial infarction and ischemia/reperfusion injury

MI is characterized by myocardial necrosis caused by acute and sustained coronary ischemia and hypoxia, and in reperfused MI, ischemia–reperfusion injury (IRI) represents a major pathophysiological component that further exacerbates tissue damage. Genetic studies suggest that some *FTO* risk alleles are associated with increased MI risk (Hubacek et al., 2016), although large GWAS meta-analysis have not consistently confirmed an association between *FTO* and coronary artery disease or MI (Nikpay et al., 2015). At the molecular level, m^6^A levels are increased in hypoxia/reoxygenation (H/R)-treated cardiomyocytes and in the hearts of mice subjected to ischemia/reperfusion (I/R) (Song et al., 2019). In parallel, *FTO* expression is generally reduced in the hearts of both humans and mice after MI (Mathiyalagan et al., 2019), and ischemic or hypoxic stress may suppress the activity of FTO, an alpha-KG-dependent dioxygenase, thereby potentially altering RNA methylation regulatory networks (Jia et al., 2011).

Most experimental studies suggest that reduced *FTO* expression is associated with aggravated myocardial injury, whereas restoration of *FTO* expression has been linked to improved cardiac outcomes. *In vitro* and *in vivo* studies indicate that *FTO* overexpression is associated with reduced apoptosis, suppressed inflammation, and improved cellular viability and energy metabolism following ischemic or hypoxic stress (Deng et al., 2021; Shen et al., 2021; Ke et al., 2022). For example, *Fto* has been reported to limit cardiomyocyte apoptosis and inflammation by enhancing the stability of yes-associated protein 1 (*Yap1*) mRNA (Ke et al., 2022). In addition, cardioprotective adaptations such as short-term fasting and chronic hypoxia have been associated with increased cardiac *FTO* expression, which correlates with enhanced ischemic or hypoxic tolerance.By contrast, inhibition of *FTO* in cardiomyocytes reduces hypoxic tolerance, further supporting a potential protective role of *FTO* in ischemic settings (Benak et al., 2024a; Hlavackova et al., 2026). Temporal changes in RNA methylation further highlight the dynamic nature of *FTO* regulation. m^6^A levels increase during both the acute ischemic phase and the reperfusion phase, whereas *FTO* expression declines significantly after ischemia; this reduction has been associated with larger infarct size and worse cardiac function. Conversely, cardiac-specific *FTO* overexpression has been associated with lower m^6^A levels and improvements in fibrosis and cardiac function (Mathiyalagan et al., 2019).

Beyond cell survival, FTO has also been implicated in post-infarction remodeling processes. Recent studies suggest that FTO may influence inflammatory microenvironment and fibrotic remodeling. For example, alpha-KG supplementation has been associated with a shift of macrophages toward an anti-inflammatory phenotype, potentially involving FTO-related RNA methylation mechanisms and janus kinase 1 (JAK1)/signal transducer and activator of transcription 3 (STAT3) signaling, along with reduced infiltration of pro-inflammatory Ly6C^+^ macrophages and improved cardiac outcomes (Lin et al., 2025). Consistent with this, FTO has been reported to be associated with reduced fibrosis and scar formation in the infarcted mouse heart (Mathiyalagan et al., 2019). Ischemia and hypoxia are accompanied by downregulation of *Fto*, whereas restoration of *Fto* expression has been associated with modulation of fibrosis- and repair-related transcripts, such as glutamyl-prolyl-tRNA synthetase (*Eprs*), along with reduced collagen deposition and improved cardiac function (Wang et al., 2024). In addition, *FTO* downregulation after MI has been associated with increased m^6^A modification of SERCA2a-encoding mRNA, reduced SERCA2a protein expression, and impaired Ca^2+^ homeostasis, whereas *FTO* upregulation has been linked to restoration of SERCA2a expression, improved calcium reuptake, and enhanced myocardial contractility (Yang et al., 2024).

However, not all studies have reached consistent conclusions. Some reports have found no significant change in FTO protein levels after H/R injury and suggest that other m^6^A regulators, such as methyltransferase-like 3 (METTL3) and ALKBH5 may play more direct regulatory roles in this process (Song et al., 2019). Taken together, current evidence suggests that FTO is involved in multiple aspects of ischemic myocardial injury and post-infarction remodeling, although its precise role remains context-dependent and may vary according to the phase of injury, experimental model, and interactions with other RNA modification regulators.

### 3.4 Myocardial fibrosis

Myocardial fibrosis is a major pathological basis of ventricular remodeling and can reduce myocardial compliance, eventually contributing to HF. Evidence shows that FTO is an important regulator of myocardial fibrosis. In models of diabetic cardiomyopathy and exercise-related myocardial fibrosis, increased cardiac m^6^A levels are often accompanied by reduced *FTO* expression; restoration of *FTO* has been associated with reduced m^6^A levels, along with attenuation of fibrosis and myocardial hypertrophy, and improves cardiac function (Ju et al., 2021; Liu et al., 2025).

Circular RNA CUGBP Elav-like family member 1 (circCELF1) has been reported to regulate the expression of Dickkopf WNT signaling pathway inhibitor 2 (DKK2), potentially through FTO-dependent m^6^A demethylation and binding to microRNA-636 (miR-636), which inhibits activation and migration of cardiac fibroblasts and reduces cardiac fibrosis (Li et al., 2022b). FTO-related regulation has also been linked to the phosphatidylinositol 3-kinase (PI3K)/protein kinase B (AKT)/glucose transporter 2 (GLUT2) pathway, the peroxisome proliferator-activated receptor (PPAR)/retinoid X receptor (RXR) pathway, and mitochondrial apoptotic signaling (Gao et al., 2022). Overall, FTO tends to show protective effects in myocardial fibrosis, although the upstream triggers and downstream targets differ across experimental models.

### 3.5 Heart failure

HF represents the end stage of many cardiovascular disorders and is characterized by impaired ventricular filling or ejection caused by structural and functional cardiac abnormalities. Major pathological features include maladaptive hypertrophy, fibrosis, and reduced contractility. It should be noted that heart failure represents a heterogeneous syndrome comprising distinct phenotypes, such as heart failure with reduced ejection fraction (HFrEF) and preserved ejection fraction (HFpEF), which differ in underlying pathophysiology (Simmonds et al., 2020). Most of the available evidence is derived from experimental models that resemble HFrEF, particularly ischemic or post-infarction models, whereas the role of FTO in HFpEF remains largely unexplored.

Previous studies show that *Fto* deficiency accelerates HF progression, as reflected by reduced ejection fraction and aggravated ventricular dilation (Berulava et al., 2020). In H/R models, *Fto* overexpression protects cardiomyocytes by suppressing apoptosis potentially through regulation of m^6^A modification (Shen et al., 2021). *Fto* is typically downregulated in failing hearts and in hypoxic cardiomyocytes, whereas restoration of *Fto* expression promotes the demethylation of transcripts involved in contractile function, increases their protein expression, and improves cardiomyocyte contractility (Mathiyalagan et al., 2019).

Mechanistic studies further suggest that reduced FTO activity increases global m^6^A levels in failing hearts from both humans and mice after MI (Mathiyalagan et al., 2019). Cardiomyocyte-specific *Fto* deficiency impairs cardiac function, whereas *Fto* overexpression delays HF progression (Berulava et al., 2020). *Fto* regulates contraction-related targets involved in calcium handling and contraction, including SERCA2a, myosin heavy chain 6/7 (MYH6/7), and ryanodine receptor 2 (RYR2) in an m^6^A-dependent manner and influences the expression of proteins including calmodulin 1 (CALM1) and SET and MYND domain-containing protein 1 (SMYD1), supporting myocardial contraction and adaptive remodeling (Mathiyalagan et al., 2019; Berulava et al., 2020). In addition to preserving contractile function, *Fto* may improve post-MI HF by promoting angiogenesis and reducing fibrosis (Mathiyalagan et al., 2019).

Recent studies have also shown that *Fto* enhances glucose uptake and glycolysis by upregulating glycolysis-related genes such as *Pgam2*, which alleviates stress-induced cardiac dysfunction in mice (Zhang et al., 2021a). Elevated *FTO* expression in the peripheral blood of patients with HF also suggests potential biomarker value (Zhang et al., 2021b). Current evidence supports a predominantly protective role of FTO in HF, although its diagnostic utility and therapeutic feasibility still require further validation.

### 3.6 Arrhythmia

Arrhythmia refers to abnormalities in heart rhythm arising from disordered electrical conduction and involves multiple mechanisms, including electrical remodeling, structural remodeling, and dysregulation of the autonomic nervous system. Previous studies show that *Fto*-knockout mice display increased heart rate, greater heart-rate variability, and enhanced susceptibility to stress-induced tachyarrhythmias, with abnormal ventricular repolarization and myocardial hypertrophy. These findings suggest that FTO is important for maintaining basal electrical stability and autonomic balance (Carnevali et al., 2014). Clinical studies have likewise shown that patients with atrial fibrillation exhibit reduced peripheral-blood *FTO* expression, which correlates with markers of metabolic dysfunction and myocardial injury, supporting the idea that low *FTO* expression may increase susceptibility to arrhythmia (Rafaqat et al., 2024).

The role of FTO in arrhythmia is not one-way. Under some pathological conditions, *Fto* upregulation may also promote atrial fibrillation. Tan et al. reported that *Fto* regulates *Kcnel* potentially through m^6^A demethylation, enhances the slow delayed rectifier potassium currents (IKs), shortens action-potential duration, and increases susceptibility to atrial fibrillation (Tan et al., 2025). Gong et al. further showed that *Fto* may upregulate *Lox* in an m^6^A-dependent manner, which promotes atrial fibrosis and raises the risk of atrial fibrillation (Gong et al., 2026). These findings suggest that the effects of FTO in arrhythmia depend on context: basal expression seems necessary for electrophysiological homeostasis, whereas pathological upregulation may increase atrial-fibrillation risk by driving electrical and structural remodeling.

### 3.7 Myocarditis

Myocarditis is an inflammatory injury of the myocardium triggered by infection, autoimmune responses, toxins, or drugs, and its core pathology involves immune dysregulation and inflammatory cascades. Evidence suggests that FTO may be involved through regulation of m^6^A modification. In sepsis models, reduced *Fto* expression is accompanied by elevated global m^6^A levels and increased expression of pro-inflammatory cytokines; restoring *FTO* suppresses inflammatory signaling and improves cardiac function (Dubey et al., 2022). In lipid-overload-associated inflammatory cardiomyopathy, *Fto* deficiency reduces fatty-acid uptake and inflammatory responses by decreasing the stability of *Cd36* mRNA (Yu et al., 2021). The net effect of *FTO* in inflammatory myocardial injury therefore seems to depend on the type of inflammatory stimulus, the metabolic background, and the dominant downstream transcripts.

**FIGURE 2:**
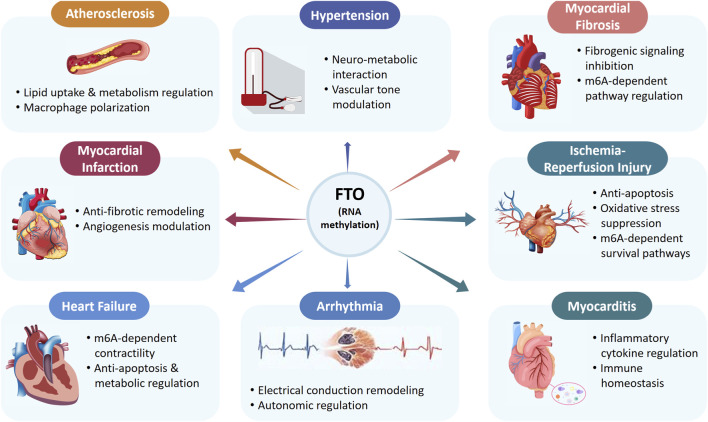
Potential roles of FTO-mediated RNA methylation in cardiovascular diseases.

These findings suggest that *FTO* influences multiple pathways involved in CVD progression through RNA demethylation (Figure 2) and may offer a potential basis for CVD treatment through m^6^A-targeted modulation using existing drugs or newly developed inhibitors.

## 4 Conclusions and future perspectives

FTO-mediated RNA methylation dynamics are increasingly recognized as potential regulators in cardiovascular diseases. However, the precise biochemical functions of FTO and the relative contributions of m^6^A versus other RNA modifications remain incompletely understood. Early GWAS and Mendelian randomization studies linked FTO mainly to obesity and cardiometabolic risk through BMI-related pathways (Fall et al., 2013). More recent work suggests that its role is not limited to BMI. FTO may also act through BMI-independent mechanisms and take part more broadly in cardiovascular pathophysiology (Äijälä et al., 2015). Its expression and function also change with age, sex, developmental stage, and the local disease environment, which suggests that FTO acts as a dynamic regulator rather than a fixed risk factor (Su et al., 2021; Semenovykh et al., 2022).

FTO has been reported to influence several biological processes, potentially through RNA methylation-related mechanisms. These include metabolic remodeling, immune and inflammatory signaling, and electrical or structural remodeling. Looking at FTO through this broader framework helps explain why previous studies have sometimes reached different conclusions and shows how complex its role is across different cardiovascular conditions.

FTO may be therapeutically relevant, but it should not be viewed as a universal target for simple inhibition or activation. Its bidirectional effects indicate that the therapeutic value of FTO depends on disease type, disease stage, cell type, and downstream transcript selectivity. Several small-molecule inhibitors targeting FTO demethylase activity have been developed and emerging evidence suggests that these compounds can influence cardiovascular and metabolic phenotypes (Huang et al., 2019; Krüger et al., 2020; Yu et al., 2021; Zhou et al., 2021). However, these findings also highlight the complexity of FTO-directed interventions, as non-selective or systemic modulation may lead to unintended or even adverse effects. In conditions such as HF, MI, myocardial fibrosis, and IRI, restoration of FTO activity appears to support cardiomyocyte metabolism, calcium handling, anti-apoptotic signaling, and repair (Mathiyalagan et al., 2019; Berulava et al., 2020; Deng et al., 2021; Shen et al., 2021; Ke et al., 2022; Yang et al., 2024). In contrast, in some settings of atrial fibrillation, inflammatory cardiomyopathy, or atherosclerosis, excessive or cell-specific FTO activation may promote electrical remodeling, endothelial inflammation, or macrophage activation (Carnevali et al., 2014; Hu et al., 2019; Gu et al., 2020a; Mo et al., 2022; Rafaqat et al., 2024). Therefore, the safest therapeutic strategy is unlikely to be systemic long-term FTO inhibition. Future approaches should aim for context-specific modulation, such as transient activation in ischemic or failing myocardium, selective inhibition in pathogenic inflammatory or vascular cell states, or transcript- or cell-targeted delivery systems. Before clinical translation, it will be essential to define therapeutic windows, disease-stage specificity, cell-specific effects, and potential off-target consequences on metabolism, immune function, and tumor biology.

Several questions still need clearer answers. The interaction between m^6^A modification and other epigenetic layers, such as DNA methylation, histone modification, and non-coding RNA regulation, is still not fully understood. RNA modifications also do not work alone, and it remains unclear how disease-specific m^6^A patterns shape phenotypic differences and disease progression in CVD. In addition, cell-specific m^6^A regulation in cardiomyocytes, endothelial cells, fibroblasts, and immune cells still needs better definition.

These considerations suggest that FTO is better regarded as a precision epitranscriptomic modulator rather than a conventional single-direction drug target. Multi-omics analysis, single-cell epitranscriptomics, spatial profiling, and detailed clinical phenotyping will help clarify the causal role of FTO in cardiovascular diseases and support its move toward precision cardiovascular medicine.
